# Phloridzin Acts as an Inhibitor of Protein-Tyrosine Phosphatase MEG2 Relevant to Insulin Resistance

**DOI:** 10.3390/molecules26061612

**Published:** 2021-03-14

**Authors:** Sun-Young Yoon, Jae Sik Yu, Ji Young Hwang, Hae Min So, Seung Oh Seo, Jung Kyu Kim, Tae Su Jang, Sang J. Chung, Ki Hyun Kim

**Affiliations:** 1 School of Pharmacy, Sungkyunkwan University, Suwon 16419, Korea; dalae1104@gmail.com (S.-Y.Y.); jsyu@bu.edu (J.S.Y.); smailemaster@naver.com (J.Y.H.); haemi9312@gmail.com (H.M.S.); wjstkz@naver.com (S.O.S.); 2 Department of Cosmetic Science, Kwangju Women’s University, Gwangju 62396, Korea; 3 School of Chemical Engineering, Sungkyunkwan University, Suwon 16419, Korea; legkim@skku.edu; 4 Department of Medicine, Dankook University, Cheonan, Chungnam 31116, Korea; jangts@dankook.ac.kr

**Keywords:** protein tyrosine phosphatases (PTPs), PTP-MEG2, phloridzin, type 2 diabetes, glucose-uptake

## Abstract

Inhibition of the megakaryocyte protein tyrosine phosphatase 2 (PTP-MEG2, also named PTPN9) activity has been shown to be a potential therapeutic strategy for the treatment of type 2 diabetes. Previously, we reported that PTP-MEG2 knockdown enhances adenosine monophosphate activated protein kinase (AMPK) phosphorylation, suggesting that PTP-MEG2 may be a potential antidiabetic target. In this study, we found that phloridzin, isolated from *Ulmus davidiana* var. *japonica*, inhibits the catalytic activity of PTP-MEG2 (half-inhibitory concentration, IC_50_ = 32 ± 1.06 μM) in vitro, indicating that it could be a potential antidiabetic drug candidate. Importantly, phloridzin stimulated glucose uptake by differentiated 3T3-L1 adipocytes and C2C12 muscle cells compared to that by the control cells. Moreover, phloridzin led to the enhanced phosphorylation of AMPK and Akt relevant to increased insulin sensitivity. Importantly, phloridzin attenuated palmitate-induced insulin resistance in C2C12 muscle cells. We also found that phloridzin did not accelerate adipocyte differentiation, suggesting that phloridzin improves insulin sensitivity without significant lipid accumulation. Taken together, our results demonstrate that phloridzin, an inhibitor of PTP-MEG2, stimulates glucose uptake through the activation of both AMPK and Akt signaling pathways. These results strongly suggest that phloridzin could be used as a potential therapeutic candidate for the treatment of type 2 diabetes.

## 1. Introduction

Protein tyrosine phosphatases (PTPs) constitute a diverse family of enzymes and control the cellular protein tyrosine phosphorylation levels associated with cell growth, metabolism, and apoptosis [1]. Loss of the regulation in protein tyrosine phosphorylation has been implicated in many diseases, including cancer, diabetes, and autoimmune disorders, suggesting that PTPs may act as potential drug targets [1,2]. Some PTPs, such as PTPN1, PTPN9, PTPN11, PTPRF, PTPRS, and dual specificity phosphatase 9 (DUSP-9), lead to type 2 diabetes associated insulin resistance through antagonism of insulin action [3,4]. Since the activation of adenosine monophosphate -activated protein kinase (AMPK) stimulates glucose uptake by promoting GLUT4 translocation, antidiabetic effects are associated with AMPK phosphorylation, suggesting that AMPK is a target for the treatment of type 2 diabetes [5,6,7,8]. We have previously shown that knockdown of the megakaryocyte protein tyrosine phosphatase 2 (PTP-MEG2, also named PTPN9) enhances AMPK phosphorylation in 3T3-L1 preadipocytes, suggesting that PTP-MEG2 can be a potential antidiabetic drug target [9]. In addition, it has been reported that PTP-MEG2 is an antagonist of liver insulin signaling and that depletion of PTP-MEG2 in the liver of diabetic (db/db) mice leads to insulin sensitization and normalization of hyperglycemia [10]. Moreover, PTP-MEG2 inhibitor has shown to enhance Akt phosphorylation in primary hepatocytes and improve insulin resistance and glucose homeostasis in diet-induced obese C57BL/6 mice, suggesting that the inhibition of PTP-MEG2 activity may be a potential therapeutic approach in treating type 2 diabetes [1]. Based on previous research, identification of the PTP-MEG2 inhibitors may be an effective strategy for treating type 2 diabetes. In this study, phloridzin was identified as a slow binding inhibitor of PTP-MEG2, suggesting that it could potentially be used as an antidiabetic drug.

Phloridzin, phloretin 2′-*O*-glucoside (sometimes also referred to as phlorizin), is a flavonoid belonging to the chemical class of dihydrochalcones and is associated with flavonoid biosynthesis [11]. Phloridzin is one of the major polyphenols found in apple (*Malus* sp.), and it is mainly present in the leaves and bark of apple trees. Low amounts of phloridzin exist in apple fruits as well. A few other species belonging to Rosaceae and Ericaceae also possess this compound, but only in low amounts [11]. Phloridzin has been widely used as an alternative medicine and possesses various pharmacological traits: scavenging effect for superoxide anions [12], inhibitory effect on lipid peroxidation [13], melanogenesis stimulatory effect through the inhibition of protein kinase C activity [14], and proliferative effect on human CD49f^+^/CD29^+^ keratinocytes [15]. In particular, the effects of phloridzin on glucose uptake and diabetes have been intensively investigated [11,16,17,18]. Phloridzin lowered the blood glucose levels and reversed *Sglt1* expression in streptozotocin-induced diabetic mice [17], and was able to inhibit the sodium-dependent glucose-linked transporter, SGLT [18]. Due to its potential applicability as a pharmacological agent, phloridzin has recently received significant attention from many research groups. However, the direct target for the antidiabetic function of phloridzin remains unknown. In this study, phytochemical analysis of the root barks of *Ulmus davidiana* var. *japonica* led to the isolation of phloridzin. We observed that phloridzin inhibited the enzymatic activity of PTP-MEG2 in vitro, indicating that phloridzin targets PTP-MEG2. We subsequently examined the effects of phloridzin in insulin-responsive cell lines such as mouse 3T3-L1 adipocytes and C2C12 muscle cells. Herein, we describe the isolation of phloridzin and its potential as an inhibitor of PTP-MEG2 relevant to insulin resistance.

## 2. Results

### 2.1. Isolation and Identification of Phloridzin

**Figure 1:**
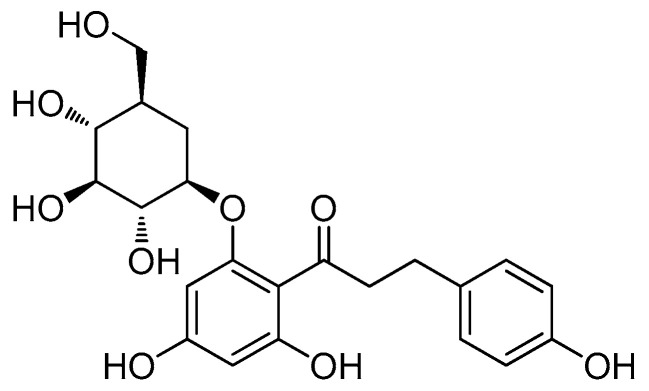
The chemical structure of phloridzin.

The crude extract of the root barks of *U. davidiana* var. *japonica* was sequentially solvent partitioned between water and organic solvents, including hexane, dichloromethane, ethyl acetate (EtOAc), and *n*-butanol of increasing polarity, yielding four fractions. Phytochemical analysis of the EtOAc-soluble fraction was performed using repeated column chromatography and semi-preparative HPLC separation, resulting in the isolation of phloridzin. The structure of phloridzin (Figure 1) was determined by comparing the ^1^H and ^13^C-NMR spectra with those previously reported in the literature [11,14,17], and by LC/MS analysis.

### 2.2. Suppression of PTP-MEG2 Enhanced AMPK Phosphorylation

**Figure 2:**
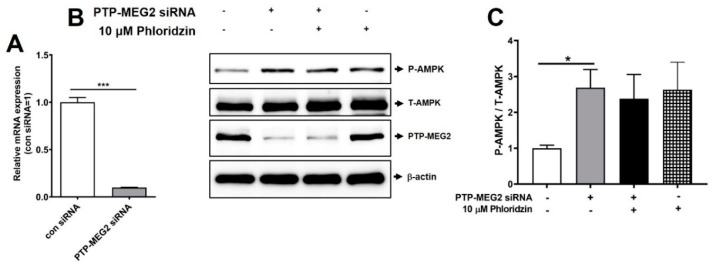
Suppression of PTP-MEG2 enhanced AMPK phosphorylation. (**A**,**B**) 3T3-L1 preadipocytes were transfected with 30 nM PTP-MEG2 siRNA or scrambled siRNA as a control (con). After 72 h, cells were lysed and analyzed by quantitative real-time PCR (**A**) or Western blotting (**B**). (**C**) Quantification of phospho-AMPK/total-AMPK using the ATTO image analysis software (CS analyzer 4). All experiments were independently repeated at 3 times. Results are presented as mean ± standard error of the mean (SEM). *** *p* < 0.001, * *p* < 0.05 compared to the control siRNA condition (con siRNA).

To identify the antidiabetic target of phloridzin, we performed PTP-MEG2 knockdown in 3T3-L1 preadipocytes using PTP-MEG2 siRNA for 72 h. Scrambled siRNA was used as a negative control. At the same time, we also incubated cells with 10 μM phloridzin for 72 h. Efficient suppression of PTP-MEG2 was confirmed by quantitative RT-PCR or Western blotting (Figure 2A,B). Consistent with our previous findings [9], we found that PTP-MEG2 knockdown significantly increased AMPK phosphorylation, suggesting that PTP-MEG2 can be a potential antidiabetic drug target (Figure 2B,C). In addition, AMPK phosphorylation was also enhanced in 10 μM phloridzin-treated cells, as compared to the control (Figure 2B,C). Furthermore, concurrent treatment with PTP-MEG2 siRNA and 10 μM phloridzin increased AMPK phosphorylation less than that of PTP-MEG2 siRNA knockdown alone, indicating that phloridzin targets PTP-MEG2 (Figure 2B,C).

### 2.3. Phloridzin Inhibited the Catalytic Activity of PTP-MEG2 In Vitro

**Figure 3:**
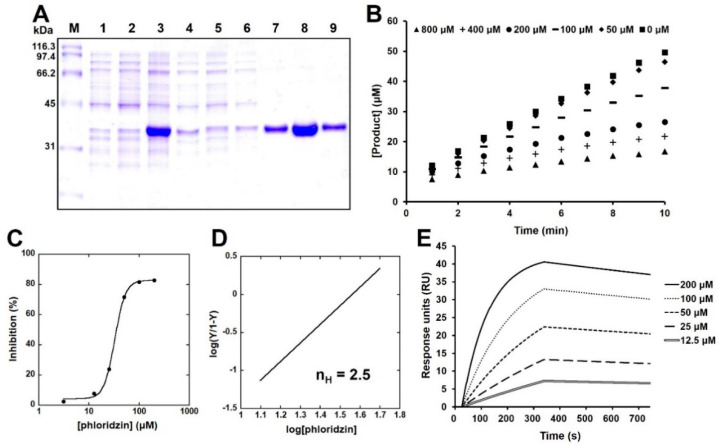
PTP-MEG2 inhibition by phloridzin. (**A**) Polyacrylamide gel electrophoresis (PAGE) analysis of PTP-MEG2 (molecular weight: 37.8 kDa). M: molecular weight marker, lane 1: total sample before induction, lane 2: supernatant before induction, lane 3: total sample after induction, lane 4: supernatant after induction, lane 5: sample passed through the column after sonication, lane 6: sample after washing the column using lysis buffer, lane 7: sample after washing the column using 10 mM imidazole, lanes 8–9: protein eluted using 100 mM imidazole. (**B**) Progress curves showing inhibition of PTP-MEG2 by phloridzin at 800 μM (▲), 400 μM (**+**), 200 μM (●), 100 μM (▬), 50 μM (♦), and 0 μM (■). (**C**) IC_50_ values estimated by fitting the sigmoid plot for % inhibition versus various concentrations of phloridzin (200, 100, 50, 25, 12.5, 3.125, and 0 μM). (**D**) Hill coefficient (nH) was calculated from the slopes of the Hill plots created according to the Hill equation. (**E**) SPR analysis was performed in running buffer (5% DMSO in PBS) using a Reichert SR7500DC system. Purified PTP-MEG2 was injected for 3 min at a flow rate of 20 μL/min. Interaction analyses were performed by injection of increasing concentrations (12.5 to 200 μM) of phloridzin at a flow rate of 30 μL/min. Association and dissociation signals were monitored for 300 and 420 s, respectively. All experiments were independently repeated at 3 times.

Next, PTP-MEG2 was overexpressed and purified using a cobalt affinity resin (Figure 3A). To measure its catalytic activity, kinetic constants of PTP-MEG2 were determined using DiFMUP as a fluorogenic PTP substrate (Table 1). Phloridzin showed better inhibition of DiFMUP hydrolysis by PTP-MEG2 than other PTPs such as PTPN1, PTPRS, PTPRF, and DUSP9 (Table 2). The progress curves of DiFMUP hydrolysis by PTP-MEG2 were linear in the absence of an inhibitor but hyperbolic progress curves were observed in the presence of phloridzin, indicating that phloridzin acted as a slow-binding inhibitor of PTP-MEG2 (Figure 3B). To estimate its half-inhibitory concentration (IC_50_), PTP-MEG2 was pre-incubated with phloridzin for 20 min and then a DiFMUP solution was added to it. The IC_50_ value of phloridzin against PTP-MEG2 was determined to be 32 ± 1.06 µM (Figure 3C). Since the Hill coefficient (*n*) obtained based on the slope of the Hill plot represents the degree of cooperativity in ligand–protein interactions (*n* > 1, positively cooperative binding; *n* = 1, noncooperative binding; *n* < 1, negative cooperative binding) [19], we estimated the extent of cooperativity between phloridzin and PTP-MEG2. We found that *n* was estimated to be 2.5 for PTP-MEG2, indicating positive cooperation in the binding of phloridzin to PTP-MEG2 (Figure 3D). Moreover, surface plasmon resonance (SPR) analysis was performed to measure the molecular interaction between phloridzin and PTP-MEG2. To this end, the association (*k* = 48.4 M^−1^s^−1^) and dissociation (*k* = 2.26 × 10^−4^ s^−1^) constants were estimated. SPR analysis revealed the specific interaction of phloridzin with PTP-MEG2, which had an equilibrium constant (*K* = *k*/*k*) value of 4.66 μM (Figure 3E). In addition, we measured the enzymatic activity of purified PTP-MEG2 by antidiabetic agents such as rosiglitazone and metformin. We found that both rosiglitazone and metformin did not inhibit the catalytic activity of PTP-MEG2 in vitro, indicating that inhibition of catalytic activity of PTP-MEG2 was phloridzin-specific among glucose-lowering agents (Supplementary Materials Figure S1).

### 2.4. Glucose Uptake Is Increased Following Phloridzin Treatment

**Figure 4:**
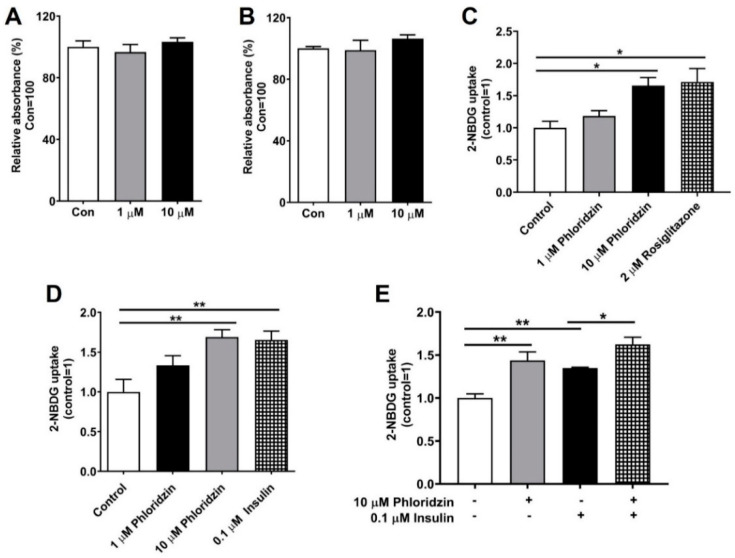
Phloridzin increases glucose uptake. (**A**,**B**) 3T3-L1 adipocytes (**A**) and C2C12 muscle cells (**B**) were incubated with the indicated concentrations of phloridzin for 2 days and cell viability was assessed by the EZ-Cytox assay kit. (**C**–**E**) Differentiated 3T3-L1 preadipocytes (**C**) and C2C12 muscle cells (**D**,**E**) were incubated for 1 h or 72 h (**E**) with 1 or 10 μM phloridzin, 0.1 μM insulin, or 2 μM rosiglitazone, and then treated with the fluorescent glucose indicator, 2-NBDG, for 30 min. For the detection of 2-NBDG, cells were washed with PBS and the fluorescence intensity was evaluated using a fluorescence microplate reader. All experiments were independently repeated at 3 times. Results are expressed as mean ± SEM. Data were analyzed using a two-tailed unpaired *t*-test. ** *p* < 0.01, * *p* < 0.05 compared to the control group.

Previously, PTP-MEG2 inhibitors have been shown to increase insulin signaling and improve glucose homeostasis in diet-induced obese mice [1]. Since glucose uptake by insulin target tissues, such as skeletal muscle, liver, and fat, plays an important role in maintaining glucose homeostasis [6], we examined the effects of phloridzin in 3T3-L1 adipocytes and C2C12 muscle cells. To assess the appropriate concentrations of phloridzin for use in cell-based studies, its cytotoxicity was studied using an EZ-Cytox assay kit. Incubation of 3T3-L1 adipocytes and C2C12 muscle cells with 1 or 10 μM phloridzin for 48 h had no effects on cell viability, indicating that these concentrations were suitable for cell treatment (Figure 4A,B). 

We next elucidated the effects of phloridzin on the glucose uptake of differentiated 3T3-L1 adipocytes and C2C12 muscle cells using the fluorescent glucose probe, 2-(*N*-(7-nitrobenz-2-oxa-1, 3-diazol-4-yl) amino)-2-deoxyglucose (2-NBDG). Cells were incubated with 1 or 10 μM phloridzin, 0.1 μM insulin (positive control), or 2 μM rosiglitazone (an antidiabetic drug used as the positive control) for 1 h. After changing the culture medium, the cells were treated with 2-NBDG for 30 min. After washing the cells with PBS, the fluorescence intensity (excitation/emission = 485/535 nm) was measured. We found that the phloridzin-treated cells had significantly enhanced fluorescence intensity as compared to the control, suggesting that phloridzin stimulated glucose uptake by differentiated 3T3L1 adipocytes as well as C2C12 muscle cells (Figure 4C,D). In addition, C2C12 muscle cells were cultured with 10 µM phloridzin or 0.1 µM insulin for 72 h and glucose uptake was assessed. We found that incubation with 10 µM phloridzin enhanced glucose uptake. Furthermore, concurrent treatment with 10 µM phloridzin and 0.1 µM insulin increased glucose uptake, as compared to cells treated with 0.1 µM insulin alone (Figure 4E). In addition, treatment with 10 μM phloridzin for 72 h induced the translocation of glucose transporter type 4 (GLUT4) in C2C12 muscle cells, similar to 0.1 µM insulin-treated effects (Supplementary Materials Figure S2). Treatment with 2 μM rosiglitazone or 0.1 μM insulin also increased the cellular glucose uptake, indicating that the fluorescent probe worked properly in our cell systems (Figure 4C–E).

### 2.5. Phloridzin Enhanced the Phosphorylation of AMPK and Akt

**Figure 5:**
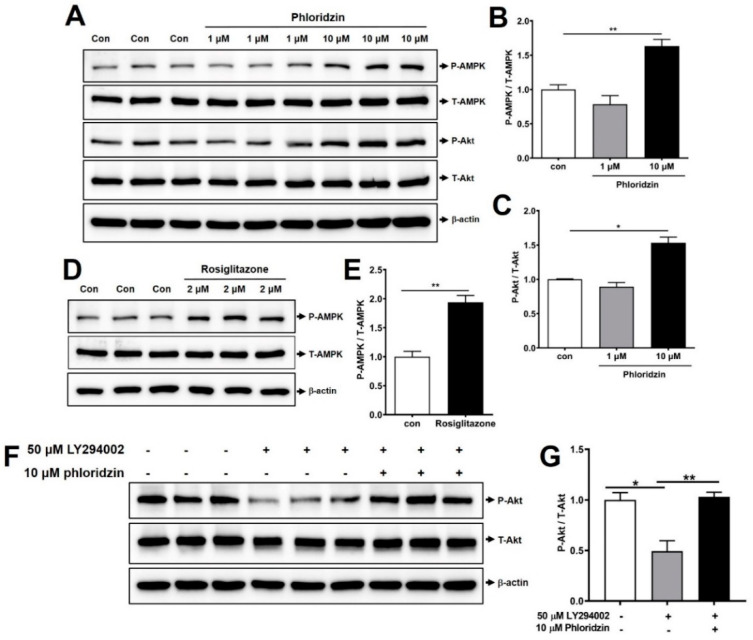
Phloridzin enhances the phosphorylation of AMPK and Akt. (**A**,**D**) Mature adipocytes were incubated with 1 or 10 μM phloridzin or 2 μM rosiglitazone. After 72 h, the cells were lysed, and Western blotting was performed using antibodies against phosphorylated AMPK, total AMPK, phosphorylated Akt, total Akt, and beta-actin. (**B**,**C**,**E**,**G**) Quantification of phospho-AMPK/total-AMPK (**B**,**E**) and phospho-Akt/total-Akt (**C**,**G**) using the ATTO image analysis software (CS analyzer 4). (**F**) 50 μM LY294002 or 10 μM phloridzin were treated in mature 3T3-L1 adipocytes. After 24 h, cells were lysed and analyzed by Western blotting. All experiments were independently repeated at 3 times. Results are presented as mean ± SEM. Data were analyzed using two-tailed unpaired *t*-test. ** *p* < 0.01, * *p* < 0.05 compared to the control group.

We next investigated the cellular mechanisms associated with increased glucose uptake. AMPK controls glucose homeostasis by regulating cellular metabolism of peripheral tissues, such as skeletal muscle, liver, and adipose tissues. Activation of AMPK leads to the stimulation of glucose uptake and lipid oxidation [7,8]. Akt signaling is associated with insulin-stimulated glucose uptake and Akt phosphorylation promotes GLUT-4 translocation and glucose uptake [20,21]. In this study, mature 3T3-L1 adipocytes were incubated with 1 or 10 μM phloridzin for 72 h and Western blotting was performed. We found that treatment with 10 μM phloridzin markedly induced AMPK phosphorylation compared to the control (Figure 5A,B).

In agreement with this result, incubation with 10 μM phloridzin significantly enhanced Akt phosphorylation compared to the control (Figure 5A,C). Rosiglitazone also remarkably increased AMPK phosphorylation, indicating that the Western blotting experiments were properly performed (Figure 5D,E). These results indicate that phloridzin increases glucose uptake through activation of both AMPK and Akt signaling pathways.

### 2.6. Phloridzin Restored LY294002-Blocked Akt Phosphorylation

We next examined the role of phloridzin in the PI3K/Akt pathway. Since LY294002, a chemical inhibitor of phosphatidylinositol-3-kinase (PI3K), reduces Akt phosphorylation, it is commonly used to study the role of the PI3K/Akt pathway [22]. In our study, we incubated mature 3T3-L1 adipocytes with 50 μM LY294002 for 24 h. Treatment with 50 μM LY294002 markedly decreased Akt phosphorylation, while 10 μM phloridzin restored LY294002-reduced Akt phosphorylation (Figure 5F,G). Our study of the PI3K inhibitor revealed that phloridzin significantly stimulated the downstream Akt signaling pathway.

### 2.7. Phloridzin Ameliorated Palmitate-Induced Insulin Resistance in C2C12 Muscle Cells

**Figure 6:**
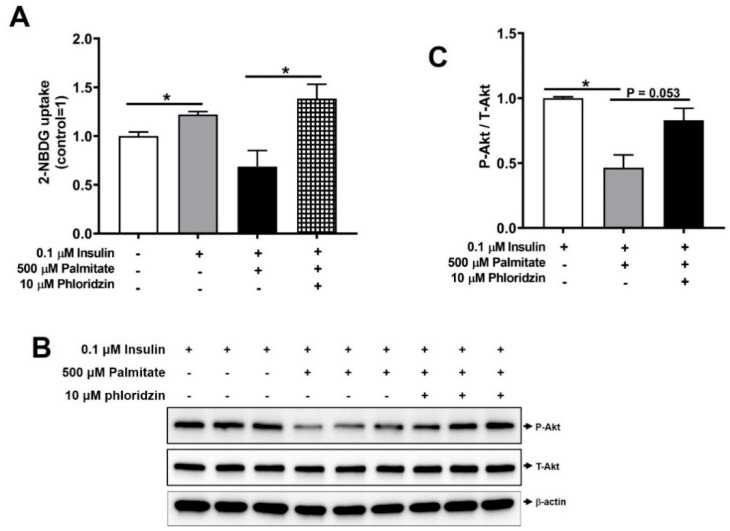
Phloridzin attenuates palmitate-induced insulin resistance in C2C12 muscle cells. (**A**,**B**) Differentiated C2C12 muscle cells were treated with 10 μM phloridzin or 500 μM palmitate for 48 h and then incubated with and without 0.1 μM insulin for 30 min. Cells were analyzed by glucose uptake (**A**) or Western blotting (**B**). (**C**) Quantification of phospho-Akt/total-Akt using the ATTO image analysis software. All experiments were independently repeated at 3 times. Results are expressed as mean ± SEM. Data were analyzed using two-tailed unpaired *t*-test. * *p* < 0.05 compared to the control group.

Next, we investigated whether phloridzin might attenuate insulin resistance in C2C12 muscle cells. Palmitate, a saturated fatty acid, has been shown to decrease insulin-stimulated glucose uptake and reduce the insulin-stimulated phosphorylation of Akt, suggesting that palmitate causes insulin resistance in C2C12 muscle cells [23,24]. When we incubated C2C12 muscle cells with 500 μM palmitate for 48 h, palmitate-treated cells decreased insulin-stimulated glucose uptake, indicating that palmitate contributes to insulin resistance (Figure 6A).

Importantly, 10 μM phloridzin attenuated palmitate-induced insulin resistance by markedly increasing glucose uptake (Figure 6A). In addition, palmitate inhibited the insulin-stimulated phosphorylation of Akt in C2C12 muscle cells using Western blotting (Figure 6B,C). Interestingly, incubation with 10 μM phloridzin ameliorated palmitate-induced insulin resistance by enhancing Akt phosphorylation (Figure 6B,C). These results indicate that phloridzin attenuates palmitate-induced insulin resistance in C2C12 muscle cells.

### 2.8. Phloridzin Did Not Enhance Lipid Accumulation

**Figure 7:**
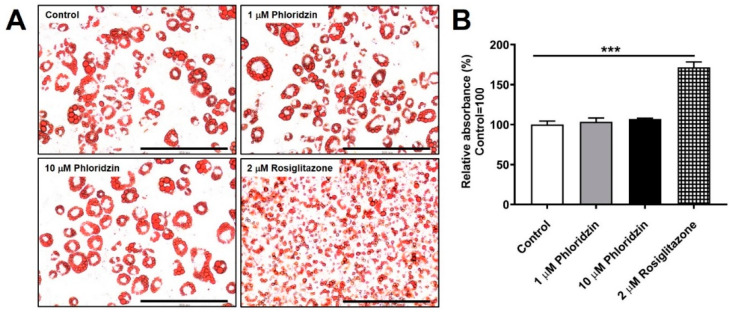
Phloridzin does not accelerate lipid accumulation. (**A**) 3T3-L1 preadipocytes were differentiated into mature adipocytes in the presence of phloridzin or rosiglitazone. The extent of lipid droplets was monitored by Oil Red O staining on day 6 of differentiation. Images of fat accumulation were acquired using an EVOS FL Imaging System. (**B**) For quantification of lipid accumulation, Oil Red O dye was eluted by incubation with isopropanol and the absorbance was measured at 490 nm using a microplate reader. All experiments were independently repeated at 3 times. Results are presented as mean ± SEM. Data were analyzed using two-tailed unpaired *t*-test. *** *p* < 0.001 compared to the control group. Scale bar: 200 μm.

We next elucidated the effect of phloridzin on lipid accumulation. 3T3-L1 preadipocytes were differentiated in the presence of phloridzin or rosiglitazone. On day 6 of differentiation, the degree of adipocyte differentiation was evaluated by Oil Red O staining. In addition, we performed quantification of lipid accumulation. To this end, Oil Red O dye was eluted by incubation with isopropanol and the absorbance was measured using a microplate reader. Adipogenesis is associated with CCAAT/enhancer-binding protein α (C/EBPα) and peroxisome proliferator-activated receptor-γ (PPARγ) [25]. Rosiglitazone, a PPARγ agonist, is an insulin-sensitizing agent with side effects such as rodents and human weight gain [26,27]. In line with this, we found that rosiglitazone significantly stimulated the formation of lipid droplets compared to the control (Figure 7A,B). 

On the contrary, phloridzin treatment did not accelerate adipocyte differentiation compared to the control, indicating that phloridzin does not enhance fat accumulation (Figure 7A,B). These results indicate that phloridzin may act as a glucose lowering agent through the PPARγ-independent signaling pathway, suggesting that phloridzin stimulates glucose uptake without significant lipid accumulation.

## 3. Discussion

Since PTPs are associated with diverse diseases, including cancer, diabetes, and autoimmune dysfunctions [2,3,4], they are suggested as next-generation drug targets. Some reports have shown that treatment with PTP-MEG2 inhibitors results in improved insulin action in both in vitro and in diet-induced obese mice [1,28], and a decrease in PTP-MEG2 in hepatocytes increases insulin sensitivity [10]. In line with our previous findings [9], we also showed that PTP-MEG2 knockdown increased AMPK phosphorylation, suggesting that PTP-MEG2 may be a potential antidiabetic drug target. Therefore, the use of PTP-MEG2 inhibitors is considered an effective strategy for the treatment of type 2 diabetes. In this study, phloridzin was identified as an inhibitor of PTP-MEG2, and, therefore, a potential anti-diabetes candidate.

Phloridzin is one of the dihydrochalcones usually found in apples and contributes to the inhibition of oxidative stress [11,17]. Some reports have shown that phloretin and phloridzin improve insulin sensitivity and enhance glucose uptake by inhibiting Cdk5 activation and corresponding PPARγ phosphorylation in differentiated 3T3L1 cells [29], and that phloretin exerts beneficial effects on the skeletal system in estrogen-deficient mice [30]. On the other hand, long-term intake of products containing high doses of phloridzin has been reported to adversely affect the musculoskeletal system in type 2 diabetic rat models [31]. Sodium–glucose co-transporter-2 (SGLT2) plays an important role in reabsorption of glucose in the kidney, and inhibition of SGLT2 has been reported as a new therapeutic strategy for diabetes [32]. Phloridzin has been shown to lack specificity for SGLT2, and phloridzin has been reported previously to improve hyperglycemia through the reduction of the blood glucose level and reverse the streptozotocin-mediated induction of SGLT1 rather than SGLT2 in the small intestine in streptozotocin-induced diabetic mice [17,32,33,34]. In addition, it has been reported that phloridzin has the proliferative effects on CD49f^+^/CD29^+^ keratinocytes via the extracellular signal-regulated kinase (ERK)-dependent mammalian target of rapamycin (mTOR) pathway [15]. However, the direct target for the antidiabetic function of phloridzin is unknown. Here, we found that phloridzin inhibited the enzymatic activity of PTP-MEG2 in vitro, indicating that phloridzin targets PTP-MEG2. Moreover, in the present study, we demonstrated that phloridzin enhances glucose uptake by differentiated 3T3-L1 adipocytes and C2C12 muscle cells through the activation of both AMPK and Akt signaling pathways.

Insulin binds to the insulin receptor on the surface of insulin-responsive tissues such as liver, adipose tissue, and skeletal muscle to induce membrane translocation of GLUT4 [35]. The activation of AMPK stimulates glucose uptake by promoting GLUT4 translocation, and reduction of GLUT4 is associated with cellular insulin resistance in diabetic animal models [5,8,36]. We also found that phloridzin induced translocation of GLUT4 in C2C12 muscle cells, suggesting that phloridzin stimulates glucose uptake by promoting GLUT4 translocation.

Insulin resistance is a major feature of type 2 diabetes and refers to insulin dysfunction in peripheral tissues, such as skeletal muscles, liver, and adipose tissues [37]. Palmitate, a saturated fatty acid, has been found to induce insulin resistance through downregulation of the insulin-stimulated phosphorylation of Akt [23,24]. Some reports have indicated that *cis*-9, *trans*-11-conjugated linoleic acid attenuates palmitate-induced insulin resistance by increasing glucose and fatty acid consumption [38], and that palmitate-induced insulin resistance was blocked by salsalate and adiponectin through inhibition of selenoprotein *p* in HepG2 cells [24]. In line with this, we also found that phloridzin ameliorated palmitate-induced insulin resistance by significantly enhancing Akt phosphorylation.

Rosiglitazone, a member of the thiazolidinedione class, is commonly used as an antidiabetic drug. Rosiglitazone has been shown to enhance glucose uptake into muscle and adipose tissue via activation of AMPK [26,39]. Rosiglitazone has adverse effects, one of which is weight gain, and has been shown to cause fat accumulation through activation of PPARγ [26,27,40]. Consistent with these findings, we found that rosiglitazone induced lipid accumulation. In contrast, phloridzin does not enhance fat accumulation compared to the control, suggesting that phloridzin improves insulin sensitivity without significant lipid accumulation.

## 4. Materials and Methods

### 4.1. Chemicals

Phloridzin (purity ≥98%, by LC/MS) was obtained from the root barks of *U. davidiana* var. *japonica* in the continuing systematic projects to isolate natural products from medicinal plants in our group. The root barks of *U. davidiana* var. *japonica* were collected from Wonhwasan-ro, Jecheon-si, Korea, in 2016, and authenticated by one of the authors (K. H. Kim). A voucher specimen (SKKU-NR 0401) of the plant was deposited at the herbarium of the School of Pharmacy, Sungkyunkwan University, Suwon, Korea. Briefly, extracts of dried root barks of *U. davidiana* var. *japonica* were prepared in 50% aqueous EtOH for 2 days at 70 °C and filtered. The filtrate was concentrated under a reduced pressure to obtain the EtOH extract, which was successively solvent-partitioned using hexane, dichloromethane, ethyl acetate (EtOAc), and *n*-butanol. The EtOAc-soluble fraction was subjected to chromatography in a Diaion HP-20 column in a gradient solvent system of MeOH (100% H O, 20% MeOH, 40% MeOH, 60% MeOH, 80% MeOH, and 100% MeOH) to yield six fractions (E0, E2, E4, E6, E8, and E10). Fraction E8 was separated by preparative reversed-phase HPLC using a gradient solvent system of MeOH-H O (45–100% MeOH) to yield six fractions (E8A–E8F). Fraction E8D was separated by preparative reversed-phase HPLC using a gradient solvent system of MeCN-H O (25–40% MeCN) to further yield six fractions (E8D1–E8D6). Phloridzin was purified from fraction E8D3 using semi-preparative HPLC using a solvent system of MeOH-H O (formic acid 0.1% (*v*/*v*)) (46% MeOH).

Phloridzin: Colorless gum; ^1^H NMR (400 MHz, CD OD): *δ* 2.90 (2H, t, *J* = 7.5 Hz, H-9), 3.41 (1H, m, H-5″), 3.45 (1H, m, H-2″), 3.46 (1H, m, H-3″), 3.47 (1H, m, H-4″), 3.48 (2H, m, H-8), 3.73 (1H, dd, *J* = 5.0 and 12.0 Hz, H-6″), 3.93 (1H, d, *J* = 12.0 Hz, H-6″), 5.06 (1H, d, *J* = 7.0 Hz, H-1″), 5.98 (1H, d, *J* = 2.0 Hz, H-5), 6.20 (1H, d, *J* = 2.0 Hz, H-3), 6.70 (2H, d, *J* = 8.5 Hz, H-2′ and H-6′), 7.08 (2H, d, *J* = 8.5 Hz, H-3′ and H-5′); ^13^C NMR (100 MHz, CD OD): *δ* 30.8 (t, C-9), 46.9 (t, C-8), 62.2 (t, C-6″), 71.3 (d, C-4″), 74.6 (d, C-2″), 78.1 (d, C-5″), 78.3 (d, C-3″), 95.2 (d, C-5), 98.3 (d, C-3), 102.1 (d, C-1″), 106.8 (s, C-1), 115.8 (d, C-2′ and C-6′), 130.2 (d, C-3′ and C-5′), 133.6 (s, C-10), 156.3 (s, C-4′), 162.2 (s, C-4), 165.6 (s, C-6), 167.5 (s, C-2), 206.5 (s, C-7); Electrospray ionization mass spectrometry (ESIMS) (positive-ion mode) *m*/*z*: 457.1 [M + Na]^+^.

### 4.2. Cell Culture

C2C12 muscle cells and 3T3-L1 preadipocytes obtained from the American Type Culture Collection (ATCC, Manassas, VA, USA) were cultured as previously described [5,25]. 3T3-L1 preadipocytes were grown in high glucose Dulbecco’s modified Eagle’s medium (DMEM; Welgene, Seoul, Republic of Korea) containing 10% bovine calf serum (BCS; Thermo Fisher Scientific, Massachusetts, USA) and an antibiotic-antimycotic solution (Welgene). C2C12 muscle cells were cultured in DMEM supplemented with 15% fetal bovine serum (FBS; Welgene) and the antibiotic-antimycotic solution. Cells were incubated at 37 °C in a 5% CO incubator.

### 4.3. Cell Differentiation

The methods used for differentiating 3T3-L1 preadipocytes and C2C12 cells were as described previously [5,25]. Once 3T3-L1 preadipocytes reached 100% confluency, they were cultured in DMEM supplemented with 10% FBS, antibiotic-antimycotic solution, 0.5 mM isobutylmethylxanthine (IBMX; Merck KGaA, Darmstadt, Germany), 1 μM dexamethasone (Sigma-Aldrich, Saint Louis, MO, USA), and 5 μg/mL insulin (Merck KGaA, Darmstadt, Germany) for 2 days. Cells were then maintained in DMEM supplemented with 10% FBS, antibiotic-antimycotic solution, and 5 μg/mL insulin for 2 days. Next, cells were incubated for 4 days in DMEM containing 10% FBS and antibiotic-antimycotic solution. When C2C12 muscle cells reached 100% confluency, they were differentiated into myotubes in DMEM supplemented with 2% horse serum (Thermo Fisher Scientific), antibiotic-antimycotic solution, and 5 μM insulin for 4 days. In all the experiments, the culture medium was replenished daily.

### 4.4. Glucose Uptake Assay

The methods used for evaluating glucose-uptake in C2C12 muscle cells and 3T3-L1 preadipocytes were as described previously [5]. Differentiated cells were grown in low-glucose DMEM (Gibco BRL) for 4 h and then incubated with phloridzin or rosiglitazone (an antidiabetic drug used as a positive control [41]) in glucose-depleted DMEM (Gibco BRL) for 1 h. The cells were treated with 5 μM of the fluorescent glucose probe 2-[N-(7-nitrobenz-2-oxa-1,3-diazol-4-yl)amino]-2-deoxyglucose (2-NBDG; Thermo Fisher Scientific) for 30 min. After washing the cells with phosphate-buffered saline (PBS, Welgene), the cell pellets were resuspended in PBS and passed through a 40 μm cell strainer. The fluorescence intensity (excitation/emission = 485/535 nm) of the cells was measured using a fluorescence microplate reader (Victor^TM^ X4, PerkinElmer, Waltham, MA, USA).

### 4.5. Overexpression and Purification of Recombinant PTPs

The methods used for the overexpression and purification of PTPs were as described previously [21]. The N-terminal His6-tagged human PTP-MEG2 and PTPN1 were transformed into *Escherichia coli* (*E. coli*) Rosetta (DE3) (Merck Millipore, Darmstadt, Germany). The human genes of PTPRS, PTPNF, and DUSP9 with both an N-terminal maltose-binding protein (MBP) and a C-terminal His6-tag were transformed into *E. coli* Rosetta (DE3). For the expression of the recombinant PTPs, cells were induced for 16 h at 18 °C using 0.1 mM isopropyl β-D-1-thiogalactopyranoside (IPTG). Cells were then harvested by centrifugation (3570× *g* for 15 min at 4 °C), washed with buffer A consisting of 50 mM Tris pH 7.5, 500 mM NaCl, 5% glycerol, 0.025% 2-mercaptoethanol, and 1 mM phenyl-methylsulfonyl fluoride (PMSF), and then lysed in buffer A by ultrasonication. After centrifugation (29,820× *g* at 4 °C for 30 min), the supernatant was incubated with a cobalt affinity resin (TALON^®^, Takara Korea, Seoul, Korea) on a shaker at 4 °C for 1 h. The resin was then washed with buffer A containing 10 mM imidazole. PTPs were eluted with buffer A supplemented with 100 mM imidazole and stored at −80 °C until further use.

### 4.6. Measurement of Enzymatic Activity and Half-Inhibitory Concentration (IC_50_) Value

The enzymatic activity of purified PTP-MEG2 was assayed using 6,8-difluoro-4-methylumbelliferyl phosphate (DiFMUP) (100 μM), a commonly used PTP substrate, as previously described [42]. To determine the *K* values, 62.5 pM of PTP-MEG2 was added to the reaction buffer (20 mM tris pH 6.0, 150 mM NaCl, 2.5 mM dithiothreitol (DTT), 0.01% Triton X-100) containing DiFMUP (800, 400, 200, 100, 50, 25, 12.5, or 6.25 μM) in 96-well plates (to a final volume of 100 μL). Fluorescence intensities were measured continuously for 10 min (excitation/emission = 355/460 nm) using a Victor^TM^ X4 multi-label plate reader (Perkin Elmer, Norwalk, CT, USA), and *K* values were determined using Lineweaver-Burk plots. To identify the mode of inhibition of phloridzin, phloridzin at concentrations of 800, 400, 200, 100, 50, and 0 μM was added to DiFMUP (2 × *K*; 326.2 μM) in the reaction buffer. After addition of 62.5 pM of PTP-MEG2, progress curves were plotted against the product concentration. To estimate IC_50_ values, various concentrations of phloridzin (200, 100, 50, 25, 12.5, 3.125, and 0 μM) were pre-incubated with 62.5 pM of PTP-MEG2 for 20 min, following which, the solution containing 326.2 μM DiFMUP was added to these mixtures. IC_50_ values were determined by fitting the sigmoid plots for the % inhibition versus various concentrations of phloridzin (KaleidaGraph, Synergy Software, Reading, Pennsylvania, USA) used. The Hill coefficient (*n*), which measures the degree of cooperativity between phloridzin and PTP-MEG2, is defined as the slope of the Hill plot created using the Hill equation [22].

### 4.7. Surface Plasmon Resonance

Surface plasmon resonance (SPR) analysis was performed in running buffer (5% dimethyl sulfoxide, DMSO in PBS) using a Reichert SR7500DC system (Reichert Technologies, Depew, NY, USA). For immobilization of PTP-MEG2, carboxymethyl dextran hydrogel (CMDH) surface sensor chips were activated with a mixture of 0.1 M EDC (N-ethyl-*N*′-(3-dimethylaminopropyl)carbodiimide) and 0.05 M NHS (N-hydroxysuccinimide) for 7 min and purified PTP-MEG2 (32 μg/mL 10 mM sodium acetate, pH 6.0) was injected for 3 min at a flow rate of 20 μL/min. Deactivation of the surface was performed using 1 M ethanolamine (pH 8.5) for 8 min. Interaction analyses were performed by injection of increasing concentrations (12.5 to 200 μM) of Phloridzin at a flow rate of 30 μL/min. Association and dissociation signals were monitored for 300 and 420 s, respectively. For the analysis, background signal recorded in reference channel was subtracted and then binding signals were fitted. Data was analyzed using Scrubber2 software (BioLogic Software, Campbell, Australia).

### 4.8. Western Blotting

Proteins were extracted using the lysis buffer containing 25 mM hydroxyethylpiperazine ethane sulfonic acid (HEPES), 150 mM NaCl, 1% Triton X-100, 10% glycerol, 5 mM EDTA, 10 mM NaF, 2 mM Na VO, and a protease inhibitor cocktail (Roche Korea, Seoul, Republic of Korea). Proteins were separated by electrophoresis using a 10% sodium dodecyl sulfate-polyacrylamide gel and transferred to a polyvinylidene fluoride membrane (Merck KGaA, Darmstadt, Germany) using a wet transfer system. Membranes were incubated overnight at 4 °C with the following primary antibodies: anti-total AMPK, anti-phosphorylated AMPK, anti-total Akt, anti-phosphorylated Akt (Cell Signaling Technology, Beverly, MA, USA), PTP-MEG2 (Santa Cruz Biotechnology, Dallas, TX, USA), and anti-beta-actin (AbFrontier, Seoul, Republic of Korea). Membranes were then probed with an anti-rabbit-IgG-horseradish peroxidase-conjugated secondary antibody (Santa Cruz Biotechnology). Antibody–antigen complexes were detected using enhanced chemiluminescence (ECL) reagents (GE Healthcare Korea, Songdo, Republic of Korea).

### 4.9. RNA Interference

Knockdown of PTP-MEG2 in 3T3-L1 preadipocytes was performed using small interfering RNAs (siRNA, Bioneer, Daejeon, Korea). Scramble siRNA was used as the negative control (Bioneer). Transfections were performed using Dharmafect (Dharmacon, GE Healthcare Korea, Songdo, Korea) according to the manufacturer’s instructions.

### 4.10. Quantitative Real-Time Polymerase Chain Reaction (qRT-PCR)

Total RNA was isolated from 3T3-L1 preadipocytes using NucleoSpin RNA Plus (Macherey-Nagel, Duren, Germany) and treated with DNase (Macherey-Nagel) to remove genomic DNA. The total RNA (1 μg) was used to synthesize cDNA with the High-Capacity Reverse Transcription kit (Applied Biosystems, Foster City, CA, USA). PCR was performed on a CFX Connect Real-Time PCR Detection System (Bio-Rad, Hercules, CA, USA) using SsoAdvanced Universal SYBR green super-mix (Bio-Rad) according to the manufacturer’s instructions. Gene expression levels of PTP-MEG2 were normalized to levels of the control gene, glyceraldehyde 3-phosphate dehydrogenase (GAPDH). Primer information is provided in Supplementary Materials Table S1.

### 4.11. Oil Red O Staining

Lipid droplets of 3T3-L1 adipocytes were assessed by Oil Red O staining, as described previously [25]. On day 6 after differentiation, cells were washed with PBS, and fixed with 4% paraformaldehyde for 15 min. The cells were then washed with PBS and stained with filtered 0.3% Oil Red O solution in isopropanol for 30 min. After washing the cells with PBS, images of fat accumulation were acquired using an EVOS FL Imaging System (Thermo Fisher Scientific Korea Ltd., Seoul, Korea). For quantification of lipid accumulation, Oil Red O dye was eluted by incubation with isopropanol and the absorbance was measured at 490 nm using a microplate reader (Victor^TM^ X4).

### 4.12. Statistical Analysis

Statistical significance (*p* < 0.05) was determined using a two-tailed unpaired *t*-test (GraphPad Software, San Diego, CA, USA). All experiments were independently repeated at 3 times. ATTO image analysis software (CS analyzer 4) was used for protein quantification. Results are presented as mean ± standard error of the mean (SEM). *** *p* < 0.001, ** *p* < 0.01, * *p* < 0.05 compared to the control group.

## 5. Conclusions

We previously reported that PTP-MEG2 knockdown enhances AMPK phosphorylation, suggesting that PTP-MEG2 may be a potential antidiabetic target [9]. Here, phloridzin was obtained from the root barks of *U. davidiana* var. *japonica* as part of our ongoing research projects to discover bioactive natural products [43,44,45,46,47,48,49], and for the first time, we found that phloridzin inhibited the catalytic activity of PTP-MEG2 in vitro, indicating that phloridzin targets PTP-MEG2. Through our cell-based studies, we demonstrated that phloridzin stimulated glucose uptake by differentiated 3T3-L1 adipocytes and C2C12 muscle cells through the activation of both AMPK and Akt signaling pathways. Importantly, phloridzin ameliorated palmitate-induced insulin resistance in C2C12 muscle cells. Moreover, we found that phloridzin did not promote fat accumulation, suggesting that phloridzin improves insulin sensitivity without significant lipid accumulation. Taken together, our results suggest that phloridzin, an inhibitor of PTP-MEG2, could be a potential therapeutic candidate for the treatment of type 2 diabetes.

## Figures and Tables

**Table 1:** Kinetic constants for 6,8-difluoro-4-methylumbelliferyl phosphate (DiFMUP )hydrolysis by PTP-MEG2. Kinetic constants of PTP-MEG2 for measuring enzyme activity were determined using DiFMUP as a fluorogenic PTP substrate.

	[E] (pM)	*K* (µM)	*V* (µMmin^−1^)	*k* (min^−1^)	*k*/*K* (µM^−1^ min^−1^)
PTP-MEG2	62.5	163.1	2.166	3.47 × 10^4^	2.12 × 10^2^

**Table 2:** Inhibition of PTPs by phloridzin treatment. PTPs were added to solutions containing 200 μM phloridzin in reaction buffer with DiFMUP (2 × *K*).

PTPs	PTPN1	PTPRS	PTP-MEG2	PTPRF	DUSP9
Inhibition (%)	39 ± 3	18 ± 1	81 ± 1	11 ± 2	13 ± 2

## Data Availability

Data is contained within the article or Supplementary Material.

## Supplementary Materials

Figure S1: Rosiglitazone and metformin do not inhibit the catalytic activity of PTP-MEG2, Figure S2: GLUT4 translocation was increased by 10 µM phloridzin. Table S1: Primer sequences used to target mouse genes, Table S2: Kinetic constants for DiFMUP hydrolysis by PTPs.

## References

[1] Zhang S., Liu S., Tao R., Wei D., Chen L., Shen W., Yu Z.H., Wang L., Jones D.R., Dong X.C. (2012). A highly selective and potent PTP-MEG2 inhibitor with therapeutic potential for type 2 diabetes. J. Am. Chem. Soc..

[2] Gurzov E.N., Stanley W.J., Brodnicki T.C., Thomas H.E. (2015). Protein tyrosine phosphatases: Molecular switches in metabolism and diabetes. Trends Endocrinol. Metab..

[3] Hendriks W.J., Pulido R. (2013). Protein tyrosine phosphatase variants in human hereditary disorders and disease susceptibilities. Biochim. Biophys. Acta.

[4] He R.J., Yu Z.H., Zhang R.Y., Zhang Z.Y. (2014). Protein tyrosine phosphatases as potential therapeutic targets. Acta Pharmacol. Sin..

[5] Kim M.S., Hur H.J., Kwon D.Y., Hwang J.T. (2012). Tangeretin stimulates glucose uptake via regulation of AMPK signaling pathways in C2C12 myotubes and improves glucose tolerance in high-fat diet-induced obese mice. Mol. Cell Endocrinol..

[6] Balasubramanian R., Robaye B., Boeynaems J.M., Jacobson K.A. (2014). Enhancement of glucose uptake in mouse skeletal muscle cells and adipocytes by P2Y6 receptor agonists. PLoS ONE.

[7] Zhou G., Sebhat I.K., Zhang B.B. (2009). AMPK activators--potential therapeutics for metabolic and other diseases. Acta Physiol..

[8] Long Y.C., Zierath J.R. (2006). AMP-activated protein kinase signaling in metabolic regulation. J. Clin. Investig..

[9] Yoon S.Y., Lee J.H., Kwon S.J., Kang H.J., Chung S.J. (2018). Ginkgolic acid as a dual-targeting inhibitor for protein tyrosine phosphatases relevant to insulin resistance. Bioorg. Chem..

[10] Cho C.Y., Koo S.H., Wang Y., Callaway S., Hedrick S., Mak P.A., Orth A.P., Peters E.C., Saez E., Montminy M. (2006). Identification of the tyrosine phosphatase PTP-MEG2 as an antagonist of hepatic insulin signaling. Cell Metab..

[11] Gosch C., Halbwirth H., Stich K. (2010). Phloridzin: Biosynthesis, distribution and physiological relevance in plants. Phytochemistry.

[12] Ridgway T., O’Reilly J., West G., Tucker G., Wiseman H. (1997). Antioxidant action of novel derivatives of the apple-derived flavonoid phloridzin compared to oestrogen: Relevance to potential cardioprotective action. Biochem. Soc. Trans..

[13] Robak J., Gryglewski R.J. (1988). Flavonoids are scavengers of superoxide anions. Biochem. Pharmacol..

[14] Shoji T., Kobori M., Shinmoto H., Tanabe M., Tsushida T. (1997). Progressive effects of phloridzin on melanogenesis in B16 mouse melanoma cells. Biosci. Biotechnol. Biochem..

[15] Lee J., Jung E., Kim Y.S., Park D., Toyama K., Date A., Lee J. (2013). Phloridzin isolated from Acanthopanax senticosus promotes proliferation of alpha6 integrin (CD 49f) and beta1 integrin (CD29) enriched for a primary keratinocyte population through the ERK-mediated mTOR pathway. Arch. Dermatol. Res..

[16] Ehrenkranz J.R., Lewis N.G., Kahn C.R., Roth J. (2005). Phlorizin: A review. Diabetes Metab. Res. Rev..

[17] Masumoto S., Akimoto Y., Oike H., Kobori M. (2009). Dietary phloridzin reduces blood glucose levels and reverses Sglt1 expression in the small intestine in streptozotocin-induced diabetic mice. J. Agric. Food Chem..

[18] Panayotova-Heiermann M., Loo D.D., Wright E.M. (1995). Kinetics of steady-state currents and charge movements associated with the rat Na^+^/glucose cotransporter. J. Biol. Chem..

[19] Stefan M.I., Le Novere N. (2013). Cooperative binding. PLoS Comput. Biol..

[20] Mackenzie R.W., Elliott B.T. (2014). Akt/PKB activation and insulin signaling: A novel insulin signaling pathway in the treatment of type 2 diabetes. Diabetes Metab. Syndr. Obes..

[21] Sharma N., Arias E.B., Bhat A.D., Sequea D.A., Ho S., Croff K.K., Sajan M.P., Farese R.V., Cartee G.D. (2011). Mechanisms for increased insulin-stimulated Akt phosphorylation and glucose uptake in fast- and slow-twitch skeletal muscles of calorie-restricted rats. Am. J. Physiol. Endocrinol. Metab..

[22] Jiang H., Fan D., Zhou G., Li X., Deng H. (2010). Phosphatidylinositol 3-kinase inhibitor(LY294002) induces apoptosis of human nasopharyngeal carcinoma in vitro and in vivo. J. Exp. Clin. Cancer Res..

[23] Feng X.T., Wang T.Z., Leng J., Chen Y., Liu J.B., Liu Y., Wang W.J. (2012). Palmitate contributes to insulin resistance through downregulation of the Src-mediated phosphorylation of Akt in C2C12 myotubes. Biosci. Biotechnol. Biochem..

[24] Jung T.W., Choi H.Y., Lee S.Y., Hong H.C., Yang S.J., Yoo H.J., Youn B.S., Baik S.H., Choi K.M. (2013). Salsalate and Adiponectin Improve Palmitate-Induced Insulin Resistance via Inhibition of Selenoprotein P through the AMPK-FOXO1alpha Pathway. PLoS ONE.

[25] Cho Y.L., Min J.K., Roh K.M., Kim W.K., Han B.S., Bae K.H., Lee S.C., Chung S.J., Kang H.J. (2015). Phosphoprotein phosphatase 1CB (PPP1CB), a novel adipogenic activator, promotes 3T3-L1 adipogenesis. Biochem. Biophys. Res. Commun..

[26] Fryer L.G., Parbu-Patel A., Carling D. (2002). The Anti-diabetic drugs rosiglitazone and metformin stimulate AMP-activated protein kinase through distinct signaling pathways. J. Biol. Chem..

[27] Kim J., Kwak H.J., Cha J.Y., Jeong Y.S., Rhee S.D., Cheon H.G. (2014). The role of prolyl hydroxylase domain protein (PHD) during rosiglitazone-induced adipocyte differentiation. J. Biol. Chem..

[28] Wang M., Li X., Dong L., Chen X., Xu W., Wang R. (2016). Virtual screening, optimization, and identification of a novel specific PTP-MEG2 Inhibitor with potential therapy for T2DM. Oncotarget.

[29] Kumar S., Sinha K., Sharma R., Purohit R., Padwad Y. (2019). Phloretin and phloridzin improve insulin sensitivity and enhance glucose uptake by subverting PPARγ/Cdk5 interaction in differentiated adipocytes. Exp. Cell Res..

[30] Lee E.J., Kim J.L., Kim Y.H., Kang M.K., Gong J.H., Kang Y.H. (2014). Phloretin promotes osteoclast apoptosis in murine macrophages and inhibits estrogen deficiency-induced osteoporosis in mice. Phytomedicine.

[31] Londzin P., Siudak S., Cegiela U., Pytlik M., Janas A., Waligora A., Folwarczna J. (2018). Phloridzin, an Apple Polyphenol, Exerted Unfavorable Effects on Bone and Muscle in an Experimental Model of Type 2 Diabetes in Rats. Nutrients.

[32] Chao E.C., Henry R.R. (2010). SGLT2 inhibition--a novel strategy for diabetes treatment. Nat. Rev. Drug Discov..

[33] Rieg T., Vallon V. (2018). Development of SGLT1 and SGLT2 inhibitors. Diabetologia.

[34] Zhao H., Yakar S., Gavrilova O., Sun H., Zhang Y., Kim H., Setser J., Jou W., LeRoith D. (2004). Phloridzin improves hyperglycemia but not hepatic insulin resistance in a transgenic mouse model of type 2 diabetes. Diabetes.

[35] Brewer P.D., Habtemichael E.N., Romenskaia I., Mastick C.C., Coster A.C. (2014). Insulin-regulated Glut4 translocation: Membrane protein trafficking with six distinctive steps. J. Biol. Chem..

[36] Yoon S.Y., Kang H.J., Ahn D., Hwang J.Y., Kwon S.J., Chung S.J. (2019). Identification of chebulinic acid as a dual targeting inhibitor of protein tyrosine phosphatases relevant to insulin resistance. Bioorg. Chem..

[37] Goldstein B.J. (2002). Insulin resistance as the core defect in type 2 diabetes mellitus. Am. J. Cardiol..

[38] Qin H., Liu Y., Lu N., Li Y., Sun C.H. (2009). cis-9,trans-11-Conjugated linoleic acid activates AMP-activated protein kinase in attenuation of insulin resistance in C2C12 myotubes. J. Agric. Food Chem..

[39] Ye J.M., Dzamko N., Hoy A.J., Iglesias M.A., Kemp B., Kraegen E. (2006). Rosiglitazone treatment enhances acute AMP-activated protein kinase-mediated muscle and adipose tissue glucose uptake in high-fat-fed rats. Diabetes.

[40] Minge C.E., Bennett B.D., Norman R.J., Robker R.L. (2008). Peroxisome proliferator-activated receptor-gamma agonist rosiglitazone reverses the adverse effects of diet-induced obesity on oocyte quality. Endocrinology.

[41] Gerstein H.C., Yusuf S., Bosch J., Pogue J., Sheridan P., Dinccag N., Hanefeld M., Hoogwerf B., Laakso M., Mohan V. (2006). Effect of rosiglitazone on the frequency of diabetes in patients with impaired glucose tolerance or impaired fasting glucose: A randomised controlled trial. Lancet.

[42] Lee S.Y., Kim W., Lee Y.G., Kang H.J., Lee S.H., Park S.Y., Min J.K., Lee S.R., Chung S.J. (2017). Identification of sennoside A as a novel inhibitor of the slingshot (SSH) family proteins related to cancer metastasis. Pharmacol. Res..

[43] Lee S., Lee D., Ryoo R., Kim J.C., Park H.B., Kang K.S., Kim K.H. (2020). Calvatianone, a Sterol Possessing a 6/5/6/5-Fused Ring System with a Contracted Tetrahydrofuran B-Ring, from the Fruiting Bodies of *Calvatia nipponica*. J. Nat. Prod..

[44] Lee S.R., Kang H.S., Yoo M.J., Yi S.A., Beemelmanns C., Lee J.C., Kim K.H. (2020). Anti-adipogenic Pregnane Steroid from a *Hydractinia*-associated Fungus, *Cladosporium sphaerospermum* SW67. Nat. Prod. Sci..

[45] Lee S., Ryoo R., Choi J.H., Kim J.H., Kim S.H., Kim K.H. (2020). Trichothecene and tremulane sesquiterpenes from a hallucinogenic mushroom *Gymnopilus junonius* and their cytotoxicity. Arch. Pharm. Res..

[46] Trinh T.A., Park E.J., Lee D., Song J.H., Lee H.L., Kim K.H., Kim Y., Jung K., Kang K.S., Yoo J.E. (2019). Estrogenic Activity of Sanguiin H-6 through Activation of Estrogen Receptor α Coactivator-binding Site. Nat. Prod. Sci..

[47] Ha J.W., Kim J., Kim H., Jang W., Kim K.H. (2020). Mushrooms: An Important Source of Natural Bioactive Compounds. Nat. Prod. Sci..

[48] Yu J.S., Li C., Kwon M., Oh T., Lee T.H., Kim D.H., Ahn J.S., Ko S.K., Kim C.S., Cao S. (2020). a unique rearranged benzoquinone-chromanone from the hawaiian volcanic soil-associated fungal strain *Penicillium herquei* FT729. Bioorg. Chem..

[49] Yu J.S., Park M., Pang C., Rashan L., Jung W.H., Kim K.H. (2020). Antifungal Phenols from *Woodfordia uniflora* Collected in Oman. J. Nat. Prod..

