# Supplementary material for: Phloridzin Acts as an Inhibitor of Protein-Tyrosine Phosphatase MEG2 Relevant to Insulin Resistance
Source: Molecules. 2021 Mar 14;26(6):1612. doi: 10.3390/molecules26061612 (PMC7998658; doi:10.3390/molecules26061612)
Supplement: Supplementary file 1 [file molecules-26-01612-s001.pdf]

Phloridzin Acts as an Inhibitor of Protein-tyrosine Phosphatase MEG2 Relevant to Insulin Resistance

Sun-Young Yoon, Jae Sik Yu, Ji Young Hwang, Hae Min So, Seung Oh Seo, Jung Kyu Kim, Tae Su Jang, Sang J. Chung, and Ki Hyun Kim

Materials and Methods

Immunofluorescence staining

C2C12 muscle cells were fixed in 4% paraformaldehyde (PFA) for 15 min at room temperature (RT), washed in PBS and incubated with blocking solution (10% normal goat serum, Life Technologies, Frederick, USA) for 1 h at RT. Next, the cells were stained with anti-GLUT4 antibody (Bioss, Massachusetts, USA) overnight at 4˚C and, after several washes, incubated with Alexa Fluor 594 goat anti-rabbit (Invitrogen, Carlsbad, CA, USA) antibody for 30 min at RT. Hoechst 33342 (Invitrogen) was used for nuclear staining. Immunofluorescence images were acquired with a Zeiss LSM 700 confocal microscope (Carl Zeiss, Jena, Germany).

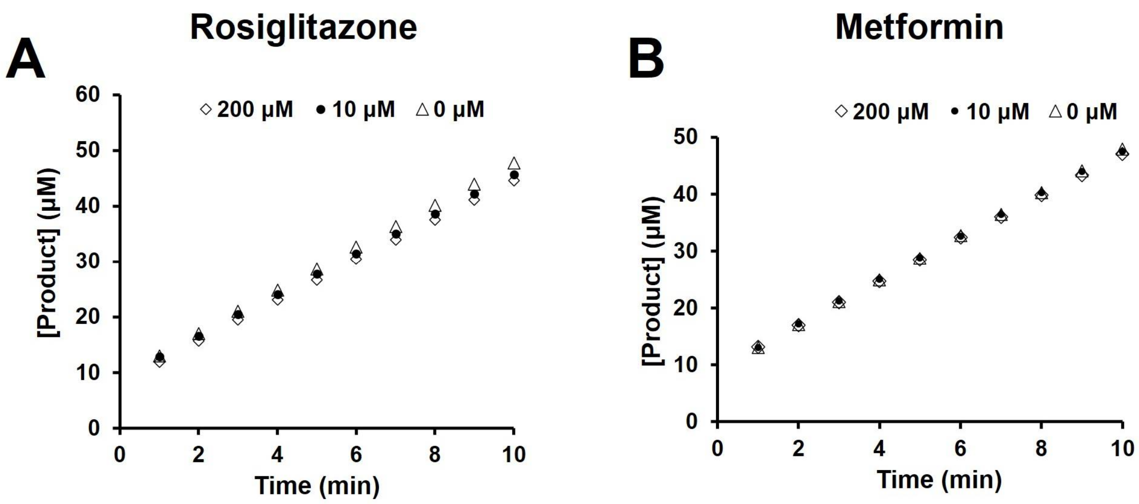

Supplementary Figure S1. Rosiglitazone and metformin do not inhibit the catalytic activity of PTP-MEG2. (**A**, **B**) Progress curves showing catalytic activity of PTP-MEG2 by rosiglitazone (**A**) or metformin (**B**) of 200 µM, 10 µM, and 0 µM.

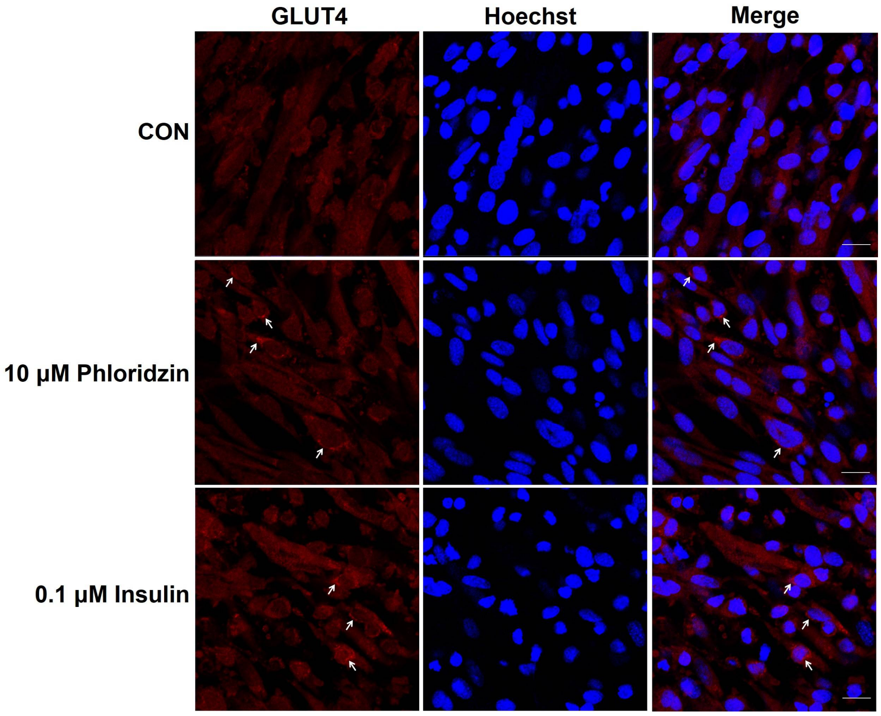

Supplementary Figure S2. GLUT4 translocation was increased by 10 μM phloridzin. Immunofluorescent staining of C2C12 muscle cells for GLUT4 (red). Nuclear staining with Hoechst 33342 (blue). Scale bar: 20 μm.

Supplementary Table S1. Primer sequences used to target mouse genes

Gene name	Primer sequence
GAPDH	
forward	GGC AAA TTC AAC GGC ACA GT
reverse	GGC GGA GAT GAT GAC CCT TT
PTP-MEG2	
forward	CCA CCC AGA TCC CTT CGA TG
reverse	CAA CAG GGT TCT CAC GAC GA

Supplementary Table S2. Kinetic constants for DiFMUP hydrolysis by PTPs

	[E] (nM)	K_M (μM)
PTPN1	0.125	22.8
PTPRS	0.25	55.8
PTPRF	0.5	50.33
DUSP9	250	34.86
